# Dysferlin at transverse tubules regulates Ca^2+^ homeostasis in skeletal muscle

**DOI:** 10.3389/fphys.2014.00089

**Published:** 2014-03-06

**Authors:** Jaclyn P. Kerr, Christopher W. Ward, Robert J. Bloch

**Affiliations:** ^1^Department of Physiology, University of Maryland School of MedicineBaltimore, MD, USA; ^2^Department of Organizational Systems and Adult Health, University of Maryland School of NursingBaltimore, MD, USA

**Keywords:** muscular dystrophy, calcium, excitation-contraction coupling, myopathy

## Abstract

The class of muscular dystrophies linked to the genetic ablation or mutation of dysferlin, including Limb Girdle Muscular Dystrophy 2B (LGMD2B) and Miyoshi Myopathy (MM), are late-onset degenerative diseases. In lieu of a genetic cure, treatments to prevent or slow the progression of dysferlinopathy are of the utmost importance. Recent advances in the study of dysferlinopathy have highlighted the necessity for the maintenance of calcium handling in altering or slowing the progression of muscular degeneration resulting from the loss of dysferlin. This review highlights new evidence for a role for dysferlin at the transverse (t-) tubule of striated muscle, where it is involved in maintaining t-tubule structure and function.

## Introduction

The class of muscular dystrophies linked to the genetic ablation or mutation of dysferlin, including Limb Girdle Muscular Dystrophy 2B (LGMD2B) and Miyoshi Myopathy (MM), are degenerative diseases of skeletal muscle that typically appear in the teen years and ultimately lead to loss of mobility. In the absence of a genetic cure, individuals with these myopathies would benefit from treatments that slow the dystrophic progression and improve quality of life. Understanding the role of dysferlin within the myofiber and how its loss affects muscle function may speed the development of therapeutics designed to prevent or ameliorate the pathogenic events that occur in its absence.

Dysferlin is a member of the ferlin subgroup, a family of proteins comprised of multiple Ca^2+^-sensitive C2 domains that are implicated in vesicle fusion, trafficking, and membrane repair (Lek et al., 2012). The seven C2 domains of dysferlin have variant affinity for Ca^2+^ and phospholipids (Davis et al., 2002; Therrien et al., 2009; Marty et al., 2013; Fuson et al., 2014) and regulate the association of dysferlin with multiple protein complexes (Huang et al., 2007; Azakir et al., 2010; Di Fulvio et al., 2011). In adult skeletal muscle cells, the early identification of dysferlin at the sarcolemma led to its assignment as a protein important for the repair of sarcolemmal damage (Bansal et al., 2003; Bansal and Campbell, 2004). However, an increasing body of evidence indicates an association of dysferlin with the transverse (t)-tubule membrane (Ampong et al., 2005; Roche et al., 2011; Flix et al., 2013; Demonbreun et al., 2014), where it is involved in maintaining Ca^2+^ homeostasis following cellular stress (Kerr et al., 2013).

Here, we review our recent evidence for dysferlin's preferential localization within the t-tubules of mature myofibers and its role in maintaining Ca^2+^ homeostasis (Roche et al., 2011; Kerr et al., 2013). Consistent with its localization at the t-tubule and its association with the L-type Ca^2+^ channel (LTCC), we showed that dysferlin contributes to the maintenance of Ca^2+^ homeostasis during mechanical stress. In dysferlin-deficient muscle fibers, acute mechanical stress disrupted Ca^2+^ homeostasis, resulting in localized t-tubule damage. As these effects were abrogated by both low external Ca^2+^ and the LTCC inhibitor diltiazem, these results are consistent with an increase of stress-dependent Ca^2+^ influx through the LTCC. Importantly, we showed that *in vivo* treatment of dysferlin-deficient mice with diltiazem provided protection from the enhanced contraction-induced damage characteristic of dysferlin-deficient muscle (Kerr et al., 2013). Taken together, our results demonstrated a novel role for dysferlin as a modulator of stress-dependent LTCC activity and identified the LTCC as a therapeutic target for LGMD2B and MM.

## Dysferlin is a T-Tubule protein

Dysferlin's large, modular structure, comprised of multiple C2 domains in tandem with structural domains common to the ferlin superfamily and a single transmembrane domain (Lek et al., 2012), makes it an attractive scaffold for structural and signaling proteins at the cytoplasmic surface of the membrane. Its role in staunching membrane damage in cultured muscle cells injured by laser illumination and its apparent translocation from internal structures to the sarcolemma led to the hypothesis that dysferlin was primarily involved in repair of the sarcolemmal membrane following Ca^2+^ influx (Bansal et al., 2003).

Additional hypotheses for dysferlin's function arose following the identification of a number of its binding partners. These binding partners include tubulin, annexins, caveolin 3, Bin1, and AHNAK, consistent with a role for dysferlin in trafficking and membrane repair (Matsuda et al., 2001; Lee et al., 2002; Lennon et al., 2003; Ampong et al., 2005; Turk et al., 2006; Huang et al., 2007; Rezvanpour and Shaw, 2009; Waddell et al., 2011; Flix et al., 2013; McDade and Michele, 2013). However, other work identified the LTCC (also referred to as the dihydropyridine receptor, or DHPR) and the ryanodine receptor (RyR) (Ampong et al., 2005; Flix et al., 2013), implicating dysferlin in Ca^2+^-dependent signaling, consistent with limited reports of dysferlin localization at or near the t-tubule during muscle maturation and stress (Roche et al., 2011; Waddell et al., 2011; Demonbreun et al., 2014).

Our evidence for dysferlin's association with the t-tubule membrane stems from improvements in the immunolabeling of frozen sections of muscle tissue and isolated muscle fibers *in vitro* (Roche et al., 2011; Kerr et al., 2013). With these improved techniques, we found a predominant association of dysferlin at the A-I junction of mature myofibers, where the triad junctions are formed between the t-tubules and the terminal cisternae of the sarcoplasmic reticulum. This localization was consistent with reports suggesting that dysferlin was involved in early t-tubule development (Klinge et al., 2010) as well as those that indicated that dysferlin could translocate to and from the t-tubules following sarcolemmal damage or extreme stretch (Klinge et al., 2007; Waddell et al., 2011). However, our results indicated that dysferlin's localization to the t-tubule was not injury-dependent and was maintained at the t-tubule following muscle maturation.

**Figure 1:**
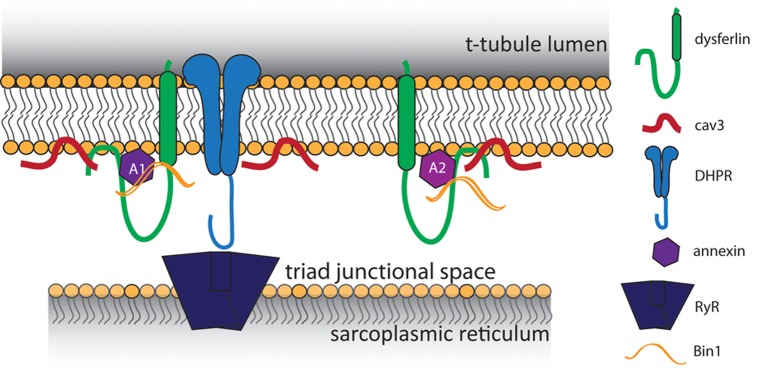
**Proposed model of t-tubule dysferlin**. Dysferlin is anchored in the t-tubule membrane by its transmembrane domain, with its extreme C-terminus exposed to the lumen of the t-tubule. In close proximity to dysferlin are proteins of the triad junction, the L-type Ca^2+^ channel (DHPR) in the t-tubule and the ryanodine receptor (RyR) in the sarcoplasmic reticulum. Caveolin 3 (Cav3) and Bin1, both important for the development of t-tubules, are known binding partners of dysferlin. Dysferlin also associates with annexins, which respond to changes in intracellular Ca^2+^ to promote wound repair.

Despite these advancements, it was impossible to determine whether dysferlin localized specifically to the t-tubule membrane using only immunofluorescence and confocal light microscopy. Therefore, we developed an expression construct that contained a specialized pH-sensitive fluorescent protein (pHluorin). When attached pHluorin to the C-terminus of dysferlin, an acute change in extracellular pH was sensed by pHluorin within 30 s, indicating its exposure to the extracellular milieu of the t-tubule lumen. In contrast, dysferlin with N-terminal pHluorin was not responsive to acute changes in external pH in this time frame (Kerr et al., 2013), consistent with the ability of the cytoplasm of mammalian striated muscles to buffer intracellular pH (Arus and Barany, 1986; Portman and Ning, 1990; Westerblad et al., 1997; Chin and Allen, 1998; Zaniboni et al., 2003; Capellini et al., 2013). We conclude from these results that dysferlin localizes in the membrane of the t-tubule, oriented with its N-terminal C2 domains in the cytoplasm and its C-terminal sequence in the lumen. Our identification of dysferlin within the t-tubule membrane is consistent with previously reported binding partners within the triad junction, noted above (Figure 1). Combined with our previous immunofluorescence studies and other reports of dysferlin's involvement in the development and maintenance of the t-tubule structure (Klinge et al., 2010; Roche et al., 2011; Waddell et al., 2011; Demonbreun et al., 2014), these studies point to a role for dysferlin in the normal function of the t-tubule.

How dysferlin arrives at the t-tubule remains an open question. Recent efforts have been directed at determining the ability of truncated dysferlin to mediate membrane repair (Azakir et al., 2012), though their focus has been on myoblasts and myotubes, rather than mature myofibers with well-organized and functional t-tubules. In that regard, dysferlin mutations causing truncation of the protein were shown to disrupt its association with Bin1 (Ampong et al., 2005), indicating that some mutations in dysferlin may reduce its association with the t-tubule and would likely affect dysferlin's function at that structure. Furthermore, the more N-terminal C2 domains of dysferlin may play an important role in its trafficking to the t-tubule, as dysferlin's C2A and C2B domains mediate microtubule binding (Azakir et al., 2010; Di Fulvio et al., 2011). Variations in dysferlin's association with the microtubule network may also affect its function at the t-tubule. Identifying the effects of disease-causing mutations of dysferlin on its targeting to t-tubules will be a critical step in uncovering the mechanisms underlying dysferlin's function in mature muscle.

## Dysferlin protects the T-Tubule from damage by mechanical stress

Our group previously demonstrated an increase in contraction-induced damage in dysferlin-deficient muscle (Roche et al., 2008, 2012). As dysregulated Ca^2+^ signaling at the t-tubule is known to underscore contraction-induced damage in Duchenne muscular dystrophy (Yeung et al., 2005; Fanchaouy et al., 2009; Shkryl et al., 2009) and dysferlin is preferentially localized within the t-tubule, we hypothesized that the t-tubules may be especially susceptible to damage in dysferlinopathy, and that a Ca^2+^ -dependent process may play a critical role in disease progression.

Our recent work implicates dysferlin in regulating Ca^2+^ signaling and homeostasis. Using a mild osmotic shock injury on isolated adult myofibers, we found increased structural disruption of the t-tubule in dysferlin-deficient myofibers. In addition, osmotic shock of dysferlin-null myofibers lead to an immediate decrease in the amplitudes of Ca^2+^ transients that was concomitant with a dramatic rise in cytosolic Ca^2+^. These effects were mitigated by blocking the LTCC with diltiazem.

Previously, we demonstrated that following damage by eccentric injury, dysferlin-deficient muscle exhibits a depressed rate of functional recovery (Roche et al., 2008; Lovering et al., 2011). Extending our *in vitro* findings, we demonstrated that diltiazem treatment *in vivo* improved the recovery of function. Examination of muscle 3 days post-injury revealed that diltiazem limited both necrosis and inflammation, and decreased the number of centrally nucleated fibers. A protection of t-tubule structure 3 h post-injury implicated diltiazem's action on the LTCC as proximate to the enhanced recovery (Kerr et al., 2013).

Although our results indicate that dysferlin protects the t-tubule from damage by mechanical stress, how it does so is unclear. Early hypotheses proposed dysferlin as a membrane repair protein, and in this capacity dysferlin may contribute to maintaining the integrity of the t-tubule membrane during mechanical stress. Consistent with this possibility, dysferlin binds to annexins A1 and A2 (Figure 1), which associate with t-tubules following injury (Waddell et al., 2011; Voigt et al., 2013). Annexins A1 and A2 are highly upregulated in patients with LGMD2B (Lennon et al., 2003), and A1 is involved in membrane repair events following membrane injury (McNeil et al., 2006; Voigt et al., 2013). An elegant study by Lek et al. (2013), demonstrated the reliance of the membrane repair mechanism on cleavage of dysferlin by calpain. In wild type cells, cellular injury results in rapid Ca^2+^ influx and cleavage of dysferlin to a synaptotagmin-like product that accumulates at the area of injury. Dysferlin's cleavage and accumulation are both blocked by inhibition of LTCC-mediated Ca^2+^ influx, indicating that Ca^2+^ influx upon injury is crucial for repair. However, as we demonstrated, influxes of Ca^2+^ in dysferlin-deficient muscle are dysregulated and sustained, resulting in secondary deficits in EC-coupling that eventually degrade the muscle fiber (Kerr et al., 2013). The pathogenic role of dysregulated Ca^2+^ signaling in other muscular dystrophies has been noted (Millay et al., 2009; Goonasekera et al., 2011).

**Figure 2:**
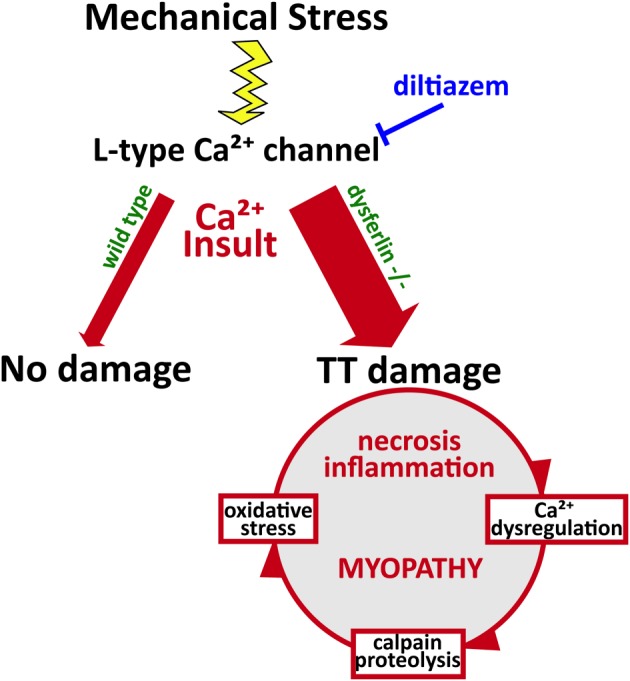
**Pathophysiology of dysferlin deficiency**. Dysferlin is hypothesized to respond to influxes of Ca^2+^ and promote wound repair of the t-tubule membrane. Mechanical stress or membrane injury results in influx of Ca^2+^, mediated by the L-type Ca^2+^ channel, and this Ca^2+^ influx does not cause significant muscle injury in wild type muscle cells. However, in the absence of dysferlin, Ca^2+^ influx to the cytosol is greatly exaggerated, disrupting Ca^2+^ homeostasis and EC-coupling. This activates a cascade of Ca^2+^-mediated events that promote further damage to the muscle fiber, including Ca^2+^-induced proteolysis and oxidative stress. Together, these processes contribute to the eventual myopathy, spurring increased necrosis and inflammation.

Recent work proposes that mechanical stress-induces the production of reactive oxygen species (ROS) by NADPH oxidase 2 (termed X-ROS). This mechano-activated ROS sensitizes the activation of mechano-sensitive Ca^2+^ channels in the t-tubule. In dystrophic muscle, X-ROS is enhanced (Prosser et al., 2011; Khairallah et al., 2012). Recently, we identified amplified X-ROS signaling in dysferlin-deficient muscle (Prosser et al., 2013; Kombairaju et al., 2014) consistent with another recent report of enhanced muscle oxidation in the same model (Terrill et al., 2013). The increased production of X-ROS in several dystrophic models suggests that it arises secondary to functional deficits linked to mutations in essential muscle genes. The contribution of X-ROS to the enhanced sensitivity to mechanical stress experienced by dysferlin-null muscle could further contribute to the muscle degeneration and myopathy that occur in dysferlinopathies (Figure 2). These pathways are all attractive targets for therapeutics, as their interactions indicate that mitigating one is likely to dampen the others.

## Conclusions

In lieu of a genetic cure, treatments that prevent or slow the progression of LGMD2B and MM are of the utmost importance. Our demonstration of altered t-tubule structure and the elevated Ca^2+^-sensitive pathways in dysferlin-null muscle is consistent with reports that both are disrupted in dysferlinopathies (Selcen et al., 2001; Campanaro et al., 2002; Suzuki et al., 2005; Demonbreun et al., 2014). Our results indicate that targeting LTCC-dependent Ca^2+^ influx is likely to have significant therapeutic benefit for patients with dysferlinopathy.

Our recent findings and those of others support a model of dysferlin as a Ca^2+^-sensitive signaling scaffold localized to the t-tubule membrane (Figure 1), that is designed to respond to changes in intracellular Ca^2+^ caused by t-tubule membrane stress and damage. We propose that this scaffold is uniquely positioned near the triad junctions of muscle, where Ca^2+^ homeostasis is tightly regulated to facilitate contraction and mediate downstream signaling cascades that maintain the normal functions of muscle. In the absence of dysferlin, the myofiber lacks the ability to maintain Ca^2+^ homeostasis during stress, resulting in abnormally high cytosolic Ca^2+^ and the activation of myriad processes that result in proteolysis and oxidative stress, and eventually, necrosis, inflammation, and the progression of the myopathy (Figure 2).

An important, unresolved question in the study of dysferlinopathy is the mechanistic underpinning for its delayed onset. Typically, symptoms do not appear until the second or third decade of life, and sometimes only much later (Klinge et al., 2008). Work by our laboratory and others has shown that, while pre-clinical animal models exhibit minor, overt functional deficits, dysferlin deficiency is clearly associated with delayed recovery from muscle injury induced by eccentric exercise (Roche et al., 2008, 2012; Biondi et al., 2013). Recent studies point to a temporal progression of altered molecular signaling and histopathology, suggesting that the phenotypic appearance in pre-clinical models and the clinical appearance of disease in patients may only be revealed after a threshold of cellular dysfunction is reached (Biondi et al., 2013). This concept is consistent with the observation that patients who participated more in sports showed more rapid disease progression (Angelini et al., 2011).

The recent re-examination of the cellular function of dysferlin highlighted in this review has provided a number of possible strategies for therapeutics. Further, these advances have expanded our understanding of dysferlin's potential roles in striated muscle. They have also stimulated new questions about the protein, how it traffics to t-tubules, and how it protects t-tubules from structural damage when muscle is stressed. Studies of the role of dysferlin's C2 domains and its effects on Ca^2+^ homeostasis and signaling may reveal an array of therapeutic options for individuals with LGMD2B and MM that will likely include both drug and genetic approaches.

## Author contributions

Jaclyn P. Kerr, Christopher W. Ward, and Robert J. Bloch wrote the manuscript.

### Conflict of interest statement

The authors declare that the research was conducted in the absence of any commercial or financial relationships that could be construed as a potential conflict of interest.
